# Parental Income Level and Risk of Developing Type 2 Diabetes in Youth

**DOI:** 10.1001/jamanetworkopen.2023.45812

**Published:** 2023-11-30

**Authors:** Fu-Shun Yen, James Cheng Chung Wei, Jia-Sin Liu, Chii-Min Hwu, Chih-Cheng Hsu

**Affiliations:** 1 Private practice, Taoyuan City, Taiwan; 2 Institute of Medicine, Chung Shan Medical University, Taichung, Taiwan; 3 Department of Medicine, Chung Shan Medical University Hospital, Taichung, Taiwan; 4 Graduate Institute of Integrated Medicine, China Medical University, Taichung, Taiwan; 5 Institute of Population Health Sciences, National Health Research Institutes, Miaoli County, Taiwan; 6 Section of Endocrinology and Metabolism, Department of Medicine, Taipei Veterans General Hospital, Taipei, Taiwan; 7 Department of Medicine, National Yang-Ming Chiao Tung University School of Medicine, Taipei, Taiwan; 8 Department of Health Services Administration, China Medical University, Taichung, Taiwan; 9 Department of Family Medicine, Min-Sheng General Hospital, Taoyuan, Taiwan; 10 National Center for Geriatrics and Welfare Research, National Health Research Institutes, Yunlin County, Taiwan

## Abstract

**Importance:**

In recent years, the global incidence of type 2 diabetes in young people has increased, especially among minoritized, Indigenous, or financially disadvantaged populations. However, few studies have examined whether poverty is associated with increased risk of youth-onset type 2 diabetes.

**Objective:**

To examine the association of family income level with the risk of youth-onset type 2 diabetes.

**Design, Setting, and Participants:**

This nationwide, population-based retrospective cohort study used data from the 2008 National Health Insurance Research Database of Taiwan, with follow-up through December 31, 2019. Participants included children and adolescents aged 0 to 19 years. Data analysis was performed from June 9, 2022, to January 16, 2023.

**Exposures:**

Family income, classified as very low, low, middle, and high.

**Main Outcomes and Measures:**

Cox proportional hazards models were used to estimate adjusted hazard ratios (aHRs) for the risks of youth-onset type 2 diabetes and all-cause mortality for all income groups vs the high-income group.

**Results:**

The cohort included a total of 5 182 893 children and adolescents (mean [SD] age, 11.2 [5.2] years; 2 477 807 girls [48.3%]). The mean (SD) follow-up duration was 9.0 (0.3) years. The incidence rates of youth-onset type 2 diabetes were 0.52 cases per 1000 person-years for the very-low-income group, 0.40 cases per 1000 person-years for the low-income group, 0.35 cases per 1000 person-years for the middle-income group, and 0.28 cases per 1000 person-years for the high-income group. Children and adolescents from very-low-income (aHR, 1.55; 95% CI, 1.41-1.71), low-income (aHR, 1.34; 95% CI, 1.27-1.41), and middle-income (aHR, 1.27; 95% CI, 1.20-1.34) families had a significantly higher hazard of youth-onset type 2 diabetes than those from high-income families. Children and adolescents from very-low-income (aHR, 2.18; 95% CI, 1.97-2.41), low-income (aHR, 1.51; 95% CI, 1.42-1.60), and middle-income (aHR, 1.22; 95% CI, 1.14-1.31) families also had a significantly higher hazard of all-cause mortality than those from high-income families. Children and adolescents who were older, female, and obese and had dyslipidemia, gout, or psychiatric disorders had a significantly higher risk of youth-onset type 2 diabetes than children without those characteristics.

**Conclusions and Relevance:**

This population-based cohort study showed that children and adolescents from very-low-income to middle-income families had a higher hazard of youth-onset type 2 diabetes and mortality than those from high-income families. Further research to reveal the factors underlying this association may improve the accuracy of identifying individuals at greatest risk for developing type 2 diabetes in youth.

## Key Points

**Question:**

Is poverty associated with youth-onset type 2 diabetes?

**Findings:**

In this nationwide, population-based cohort study of more than 5 million children and adolescents in Taiwan, those from a family with very low, low, or middle income had a significantly higher hazard of youth-onset type 2 diabetes than those from high-income families. Children and adolescents from very low, low, and middle income families also had a significantly higher hazard of all-cause mortality than those from high-income families.

**Meaning:**

These findings suggest that low family income is associated with increased risk of type 2 diabetes and all-cause mortality among children and adolescents; further research to reveal the factors underlying this association may improve the accuracy of identifying individuals at greatest risk for developing type 2 diabetes in youth.

## Teaser

This cohort study examines the association of family income level with the risk of youth-onset type 2 diabetes among children and adolescents in Taiwan.

## Introduction

Type 2 diabetes usually occurs after the age of 50 to 60 years.^1,2^ However, youth-onset type 2 diabetes, also known as juvenile or adolescent type 2 diabetes, occurs most often between the ages of 10 and 20 years.^3^ Type 1 diabetes is the main type of diabetes in people younger than 20 years. However, in recent years, the incidence and prevalence of type 2 diabetes in adolescents have increased.^4,5,6^ Reports^6,7^ have found a higher prevalence of type 2 diabetes than type 1 diabetes in young people aged 10 to 20 years in some countries and certain racial and ethnic groups. Young people with a diagnosis of type 2 diabetes will live with the condition for a longer time than those who receive a diagnosis in adulthood.^4,5^

However, youth with type 2 diabetes may be busy with schoolwork and often miss regular appointments and medications.^3,5^ They may be in a rebellious stage of adolescence and may feel pressured by families and schools.^3,8^ Insulin secretion in adolescents with type 2 diabetes tends to decline rapidly, making them susceptible to failure of oral hypoglycemic therapy.^5,9^ Therefore, young people with type 2 diabetes often have suboptimal glycemic control, making them vulnerable to complications and premature death.^3,4,5^ Identifying patients at high risk for developing adolescent type 2 diabetes is crucial.

A US study^6^ found that type 2 diabetes in youth was more prevalent among those from historically minoritized racial groups than among White individuals. In other countries, reports have found that youth-onset type 2 diabetes is more prevalent in racially oppressed groups, Indigenous, or financially deprived populations than in less-disadvantaged populations,^5,10^ with the common characteristic being poverty.^11^ However, these are broad epidemiological findings and inferences. Although poverty has been linked to the development of adult type 2 diabetes,^1,12^ few studies have examined the association of poverty with the risk of youth-onset type 2 diabetes.^3,5^ Therefore, we conducted a population-based cohort study to compare the association of different family incomes with the hazard of youth-onset type 2 diabetes in a nation with universal health coverage.

## Methods

### Study Population and Data Source

This study was conducted in accordance with the tenets of the Declaration of Helsinki.^13^ The institutional review board of the National Health Research Institutes approved this study. Participant and practitioner information were deidentified and encrypted before release to protect individual privacy. The study received a waiver of informed consent from the institutional review board. This report follows the Strengthening the Reporting of Observational Studies in Epidemiology (STROBE) reporting guidelines for observational studies.

Participants in this study were identified from the National Health Insurance Research Database (NHIRD). In 1995, Taiwan established a national health insurance program to provide health care to its citizens. National Health Insurance is a compulsory program with the government as the sole purchaser. The public pays a small premium amount, while the government and the client pay the rest. Thus, in the year 2000, approximately 99% of the 23 million people in the country were enrolled in this health insurance scheme.^14^ Information on people’s medical appointments, including their address, age, sex, diagnoses, procedures, prescriptions, and outpatient and inpatient care details, are recorded in the NHIRD. The *International Classification of Diseases, Ninth Revision (ICD-9)* and *International Statistical Classification of Diseases, Tenth Revision, Clinical Modification (ICD-10-CM)* are used for diagnosis. The NHIRD is linked to the National Death Registry to verify death information.

This cohort study included children and adolescents aged 0 to 19 years from the 2008 NHIRD full population. Exclusion criteria were age 20 years or older, diagnosis of type 1 or type 2 diabetes, gestational diabetes, or dialysis treatment (eTable 1 in Supplement 1).^15^ We divided the children and adolescents into 3 age groups (0-6 years, 7-13 years, and 14-19 years) to describe the basic characteristics of this study population. We also controlled for sex, overweight (*ICD-9* and *ICD-10-CM* coding for overweight and body mass index [calculated as weight in kilograms divided by height in meters squared] 25-29), obesity (*ICD-9* and *ICD-10-CM* coding for obesity, obesity complicating diseases, and body mass index 30-39), severe obesity (*ICD-9* and *ICD-10-CM* coding for severe obesity, obesity undergoing bariatric surgery, and body mass index ≥40), smoking status, alcohol-related disorders, hypertension, gout, psychiatric disorders (including mental disorders, schizophrenia, mood disorders, delusional disorders, psychosis, and pervasive developmental disorders), and Charlson Comorbidity Index scores (see eTable 1 in Supplement 1 for related definitions).^16^ Information on comorbidities and Charlson Comorbidity Index scores was obtained from the 2007 NHIRD records. To increase the diagnostic validity of comorbidities, we assumed at least 2 outpatient visits or 1 inpatient diagnosis.

### Economic Status

The economic status of the children and adolescents was divided into 4 groups according to their family’s monthly income using data from the NHIRD (incomes are shown as US dollars throughout; as of October 31, 2023, US $1 = 32.47 New Taiwan dollars): very low (ie, those recognized by the local government as living below the lowest living index, <$480), low (<$733; Taiwan’s minimum monthly wage in 2009 was $733), middle ($733-$1499), and high (≥$1500). The National Health Insurance administration waives premiums and copayments for very-low-income Medicare beneficiaries.

### Main End Points

Youth-onset type 2 diabetes and all-cause mortality were the main end points of this study. To ensure diagnostic accuracy, we defined children and adolescents as having type 2 diabetes if they had 3 or more outpatient visits or 1 or more inpatient admission for type 2 diabetes within 1 year. The *ICD-9* and *ICD-10-CM* coding algorithm for identifying type 2 diabetes was validated by a previous Taiwanese study,^17^ with sensitivity of 90.9% and a positive predictive value of 92.0%. To reduce the misclassification of type 1 diabetes as type 2 diabetes, we excluded those taking insulin therapy within 3 months of diabetes diagnosis and continuing use or those receiving treatment in an emergency department or hospital for diabetic ketoacidosis (eAppendix in Supplement 1). All-cause mortality was determined from the death certificate and was verified with the National Death Registry.

### Statistical Analysis

We performed the data analysis from June 9, 2022, to January 16, 2023. We used the χ^2^ test to determine statistical differences between categorical variables and the Student *t* test for continuous variables. The incidence rate of type 2 diabetes or mortality during the follow-up period was estimated as the number of outcomes per 1000 person-years. Person-years were calculated as the time elapsed from the date of cohort entry (January 1, 2008) to the date of development of outcomes, withdrawal from the NHI program, or the end of follow-up (December 31, 2019), whichever came first.

Cox proportional hazards models were used to estimate adjusted hazard ratios (aHRs) for the development of youth-onset type 2 diabetes and all-cause mortality in the 3 lower income groups compared with the high-income group. The proportional hazards assumption was tested using the Schoenfeld residuals test and complementary log-log plots. The covariates used for adjustment in this study included clinical (age, sex, obesity, and smoking), medical (alcohol disorders, hypertension, gout, psychiatric disorders, and Charlson Comorbidity Index scores), and health care utilization (number of outpatient visits per year) related factors. We used this method to compare the hazard of inpatient, outpatient, emergency, and total diagnoses of youth-onset type 2 diabetes among adolescents in these 4 family income groups. Multiple Cox regression was used to estimate aHRs and 95% CIs. The 95% CI for the aHR was calculated assuming that the aHR followed a Poisson distribution. Statistical significance was defined as a 2-tailed *P* < .05. SAS statistical software version 9.4 (SAS Institute) and Stata statistical software version 16.1 (StataCorp) were used for statistical analyses.

Kaplan-Meier methods and log-rank tests were used to examine the cumulative incidence of all and inpatient, outpatient, or emergency diagnoses of youth-onset type 2 diabetes among adolescents in these 4 income groups. Subgroup analyses were performed for the risk of youth-onset type 2 diabetes and all-cause mortality among 4 family income groups associated with age, sex, overweight, obesity, severe obesity, smoking, alcohol, hypertension, dyslipidemia, gout, and psychiatric disorders. For secondary and subgroup analyses, the potential for type I error due to multiple comparisons requires that the significance threshold be adjusted to *P* < .005. We performed an additional analysis by restricting the study participants to those aged 7 to 19 years and assessed the outcomes of youth-onset type 2 diabetes and hospitalization for diabetes complications among the 4 groups of children and adolescents.

## Results

### Basic Characteristics

From the 2008 Taiwan NHIRD, we identified 5 182 893 children and adolescents aged 0 to 19 years (mean [SD] age, 11.2 [5.2] years; 2 477 807 girls [48.3%]). After exclusions, there were 131 423 adolescents from very-low-income families, 2 915 352 adolescents from low-income families, 1 377 257 adolescents from middle-income families, and 752 326 adolescents from high-income families (eFigure in Supplement 1). The mean (SD) family monthly income was $44 ($2) for the very-low-income group, $529 ($259) for the low-income group, $1093 ($225) for the middle-income group, and $2112 ($653) for the high-income group (Table 1). The children and adolescents in the very-low-income group were older, more obese, and had more psychiatric disorders than those in the other 3 groups. The median (SD) follow-up period was 9.0 (0.3) years.

**Table 1.** Characteristics of Children and Adolescents Aged 0 to 19 Years in 2008 in Taiwan by Family Income

Characteristic	Participants, No. (%)				*P* value^a^
	Very low income (n = 131 423)	Low income (n = 2 915 352)	Middle income (n = 1 377 257)	High income (n = 752 326)	
Age group, y					
0-6	18 533 (14.1)	649 947 (22.3)	407 811 (29.6)	180 956 (24.1)	<.001
7-13	50 237 (38.2)	1 100 942 (37.8)	503 607 (36.6)	296 033 (39.3)	<.001
14-19	62 653 (47.7)	1 164 463 (39.9)	465 839 (33.8)	275 337 (36.6)	<.001
Mean (SD)	12.4 (4.8)	11.3 (5.3)	10.3 (5.5)	10.9 (5.2)	<.001
Sex					
Male	65 901 (50.1)	1 521 510 (52.2)	718 485 (52.2)	392 655 (52.2)	<.001
Female	65 522 (49.9)	1 393 842 (47.8)	658 772 (47.8)	359 671 (47.8)	
Family monthly income, mean (SD), $US^b^	44 (2)	529 (259)	1093 (225)	2112 (653)	<.001
Comorbidity					
Overweight	19 (<0.1)	352 (<0.1)	179 (<0.1)	101 (<0.1)	.66
Obesity	226 (0.2)	2498 (0.1)	1232 (0.1)	712 (0.1)	<.001
Severe obesity	25 (<0.1)	385 (<0.1)	168 (<0.1)	94 (<0.1)	.20
Smoking	7 (<0.1)	116 (<0.1)	46 (<0.1)	9 (<0.1)	.002
Alcohol-related disorders	20 (<0.1)	91 (<0.1)	33 (<0.1)	17 (<0.1)	<.001
Hypertension	121 (0.1)	1576 (0.1)	708 (0.1)	373 (<0.1)	<.001
Gout	146 (0.1)	2950 (0.1)	1247 (0.1)	622 (0.1)	<.001
Psychiatric disorders	961 (0.7)	12 353 (0.4)	6458 (0.5)	5287 (0.7)	<.001
Charlson Comorbidity Index score, mean (SD)	1.1 (0.3)	1 (0.3)	1 (0.2)	1 (0.2)	<.001
Frequency of outpatient visits per year per person, mean (SD)	30.6 (28.6)	33.5 (29.8)	37.7 (31.2)	36.1 (30.4)	<.001

^a^ *P* values reflect the probability of testing indifference in comparison of the very-low-income, low-income, and moderate-income groups with the high-income group. The high-income group is the reference.

^b^ As of October 31, 2023, US $1 = 32.47 New Taiwan dollars.

### Main Outcomes

The incidence rates of total youth-onset type 2 diabetes were 0.52 cases per 1000 person-years for the very-low-income group, 0.40 cases per 1000 person-years for the low-income group, 0.35 cases per 1000 person-years for the middle-income group, and 0.28 cases per 1000 person-years for the high-income group (Table 2). Compared with the high-income group, the aHRs for risk of youth-onset type 2 diabetes were 1.55 (95% CI, 1.41-1.71) for the very-low-income group, 1.34 (95% CI, 1.27-1.41) for the low-income group, and 1.27 (95% CI, 1.20-1.34) for the middle-income group. The aHRs for risk of inpatient diagnosed youth-onset type 2 diabetes were 2.11 (95% CI, 1.65-2.69) for the very-low-income group, 1.66 (95% CI, 1.44-1.91) for the low-income group, and 1.42 (95% CI, 1.21-1.66) for the middle-income group vs the high-income group. The aHRs for the risk of receiving a diagnosis of youth-onset type 2 diabetes in the emergency department were 2.00 (95% CI, 1.61-2.48) for the very-low-income group, 1.57 (95% CI, 1.38-1.77) for the low-income group, and 1.38 (95% CI, 1.20-1.58) for the middle-income group vs the high-income group. The aHRs for the risk of outpatient diagnosed youth-onset type 2 diabetes were 1.47 (95% CI, 1.32-1.64) for the very-low-income group, 1.30 (95% CI, 1.23-1.37) for the low-income group, and 1.24 (95% CI, 1.17-1.32) for the middle-income group vs the high-income group.

**Table 2.** Risk of All-Cause Mortality and Diabetes Incidence Among Children and Adolescents Aged 0 to 19 Years in 2008 in Taiwan by Family Income Group

Variable and family income group	Cases, No.	Incidence rate, cases/1000 person-years	Crude model		Adjusted model^a^	
			HR (95% CI)	*P* value	HR (95% CI)	*P* value
All-cause mortality						
Very low	546	0.52	2.29 (2.07-2.53)	<.001	2.18 (1.97-2.41)	<.001
Low	7708	0.33	1.52 (1.43-1.61)	<.001	1.51 (1.42-1.60)	<.001
Middle	2735	0.25	1.17 (1.10-1.26)	<.001	1.22 (1.14-1.31)	<.001
High	1263	0.21	1 [Reference]	NA	1 [Reference]	NA
Total diabetes incidence						
Very low	544	0.52	1.69 (1.54-1.87)	<.001	1.55 (1.41-1.71)	<.001
Low	9283	0.40	1.34 (1.27-1.41)	<.001	1.34 (1.27-1.41)	<.001
Middle	3828	0.35	1.22 (1.15-1.29)	<.001	1.27 (1.20-1.34)	<.001
High	1685	0.28	1 [Reference]	NA	1 [Reference]	NA
Diabetes diagnosed during inpatient treatment						
Very low	94	0.09	2.24 (1.76-2.85)	<.001	2.11 (1.65-2.69)	<.001
Low	1474	0.06	1.62 (1.41-1.87)	<.001	1.66 (1.44-1.91)	<.001
Middle	562	0.05	1.38 (1.18-1.61)	<.001	1.42 (1.21-1.66)	<.001
High	219	0.04	1 [Reference]	NA	1 [Reference]	NA
Diabetes diagnosed in emergency department						
Very low	115	0.11	2.12 (1.71-2.63)	<.001	2.00 (1.61-2.48)	<.001
Low	1852	0.08	1.56 (1.38-1.77)	<.001	1.57 (1.38-1.77)	<.001
Middle	738	0.07	1.35 (1.18-1.54)	<.001	1.38 (1.20-1.58)	<.001
High	296	0.05	1 [Reference]	NA	1 [Reference]	NA
Diabetes diagnosed during outpatient treatment						
Very low	450	0.43	1.61 (1.45-1.79)	<.001	1.47 (1.32-1.64)	<.001
Low	7809	0.34	1.29 (1.22-1.37)	<.001	1.30 (1.23-1.37)	<.001
Middle	3266	0.30	1.20 (1.12-1.27)	<.001	1.24 (1.17-1.32)	<.001
High	1466	0.24	1 [Reference]	NA	1 [Reference]	NA

Abbreviations: HR, hazard ratio; NA, not applicable.

^a^ Model was adjusted age, sex, overweight, obesity, severe obesity, smoking, alcohol, hypertension, dyslipidemia, gout, psychiatric disorders, Charlson Comorbidity Index scores, and frequency of outpatient visits per year.

**Figure 1.**
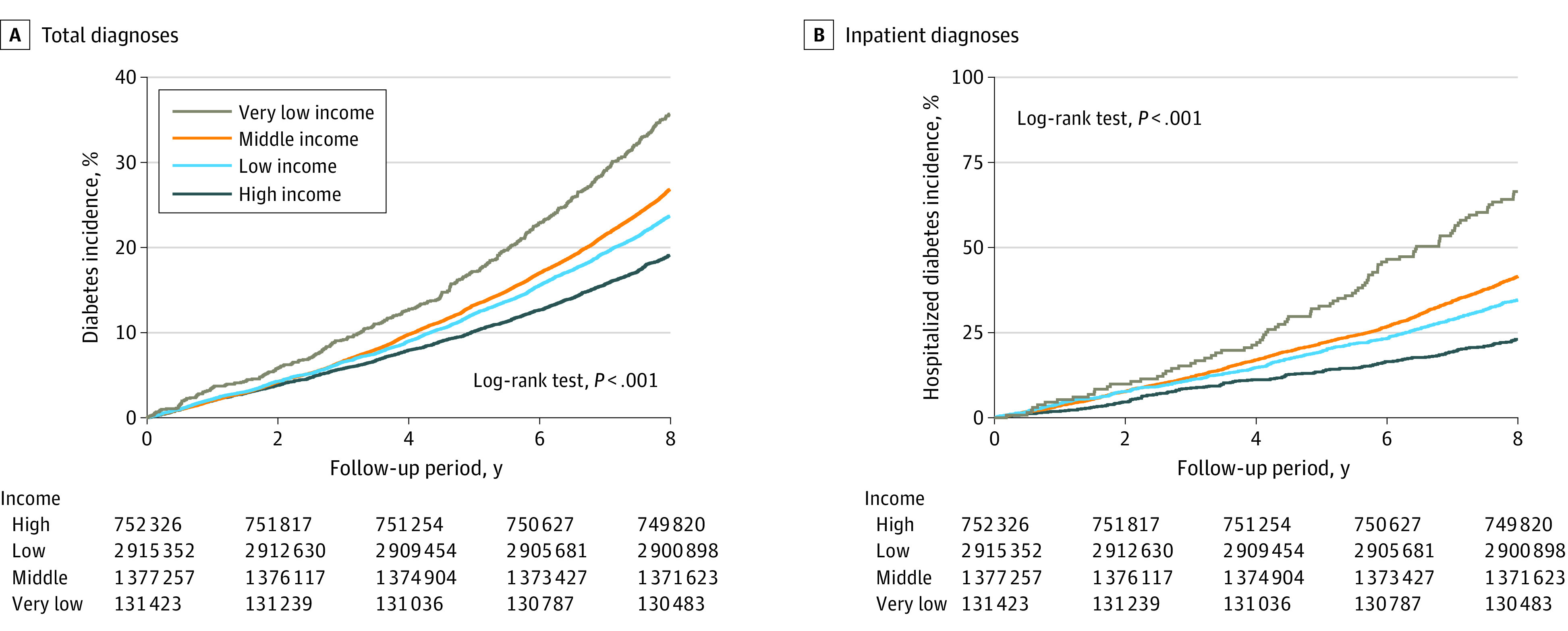
Kaplan-Meier Curves for the Incidence of Total and Inpatient Diagnosis of Type 2 Diabetes in Youth From Different Income Groups Graphs show incidence rates for total (A) and inpatient diagnosis (B) of type 2 diabetes. The *P* value in the log-rank tests compares the very low, low, and moderate income groups with the high income group.

**Figure 2.**
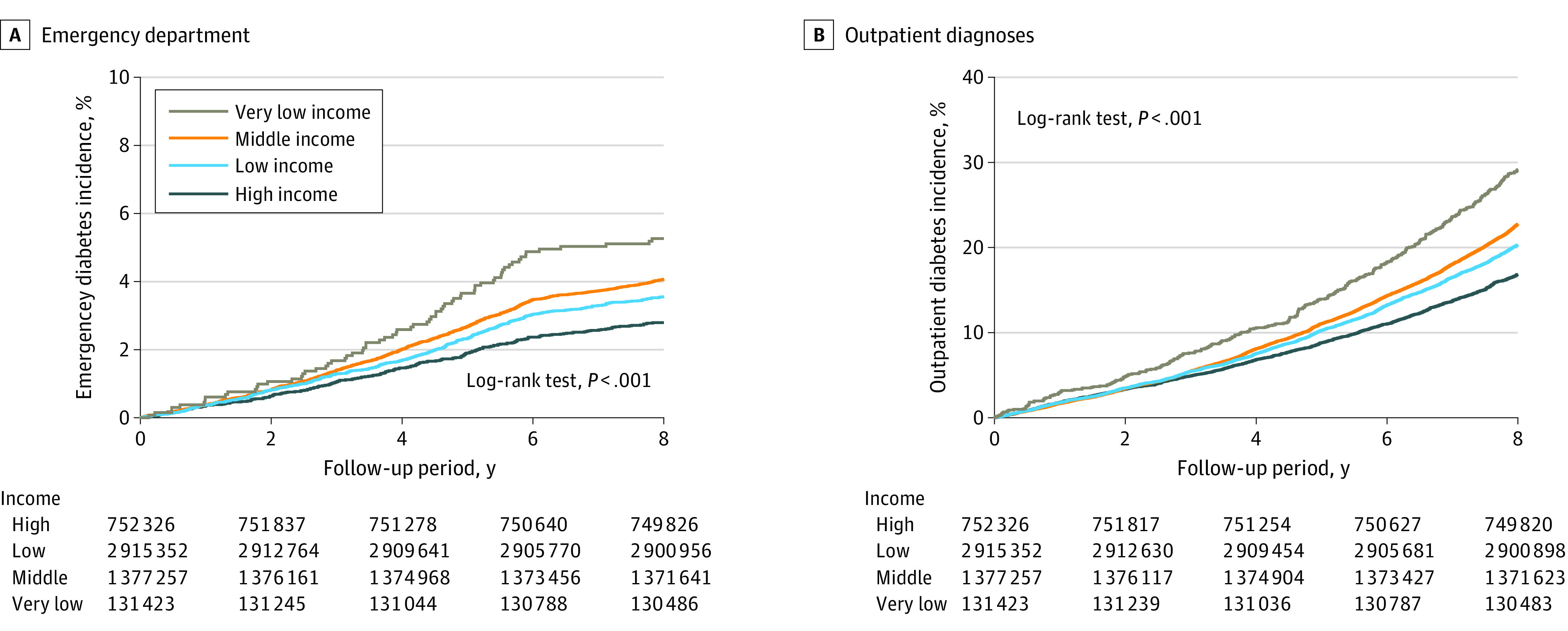
Kaplan-Meier Curves for the Incidence of Emergency Department and Outpatient Diagnosis of Type 2 Diabetes in Youth From Different Income Groups Graphs show incidence rates for emergency department (A) and outpatient (B) diagnosis of type 2 diabetes. The *P* value in the log-rank tests compares the very low, low, and moderate income groups with the high income group.

The incidence of all-cause mortality was 0.52 cases per 1000 person-years in the very-low-income group, 0.33 cases per 1000 person-years in the low-income group, 0.25 cases per 1000 person-years in the middle-income group, and 0.21 cases per 1000 person-years in the high-income group (Table 2). The aHRs for the risk of all-cause mortality were 2.18 (95% CI, 1.97-2.41) for the very-low-income group, 1.51 (95% CI, 1.42-1.60) for the low-income group, and 1.22 (95% CI, 1.14-1.31) for the middle-income group vs the high-income group. Adolescents from low-income families had a significantly higher cumulative incidence of total youth-onset type 2 diabetes (Figure 1A), inpatient diagnosed youth-onset type 2 diabetes (Figure 1B), emergency department diagnosed youth-onset type 2 diabetes (Figure 2A), and outpatient diagnosed youth-onset type 2 diabetes (Figure 2B) than those from high-income families.

### Subgroup Analyses

Among the different subgroups of children and adolescents, in addition to family income, adolescents who were older, female, obese, and had dyslipidemia, gout, and psychiatric disorders had a significantly higher hazard of youth-onset type 2 diabetes than those without these characteristics. Those who were older and male and had alcohol-related disorders, hypertension, dyslipidemia, and psychiatric disorders had a significantly higher hazard of all-cause mortality (eTable 2 in Supplement 1).

### Additional Analyses

When we restricted the analysis to children and adolescents aged 7 to 19 years, the incidence rates of total youth-onset type 2 diabetes were 0.55 cases per 1000 person-years for the very-low-income group, 0.37 cases per 1000 person-years for the low-income group, 0.29 cases per 1000 person-years for the middle-income group, and 0.24 cases per 1000 person-years in the high-income group (eTable 3 in Supplement 1). The aHRs for the risk of youth-onset type 2 diabetes were 2.18 (95% CI, 1.96-2.43) for the very-low-income group, 1.51 (95% CI, 1.42-1.62) for the low-income group, and 1.25 (95% CI, 1.16-1.34) for the middle-income group vs the high-income group. The aHRs for risk of hospitalization for diabetes complications were 2.33 (95% CI, 1.81-3.01) for the very-low-income group, 1.81 (95% CI, 1.54-2.11) for the low-income group, and 1.40 (95% CI, 1.17-1.67) for the middle-income group vs the high-income group.

## Discussion

The present cohort study found that children and adolescents with lower family incomes had a significantly higher hazard of youth-onset type 2 diabetes and all-cause mortality than those with higher family incomes. This pattern was consistent across outpatient, emergency department, and inpatient diagnoses of type 2 diabetes in adolescents. In particular, young people from very-low-income families were at the highest hazard of these outcomes. In addition to family income, children and adolescents with older age, dyslipidemia, and psychiatric disorders demonstrated a significantly higher hazard of youth-onset type 2 diabetes and all-cause mortality. We recommend more health education and regular screenings for adolescents from low-income families to reduce the future risk of developing youth-onset type 2 diabetes.

A US national surveillance study^6^ from 2009 to 2012 showed that non-Hispanic Black and Native American youth aged 10 to 20 years were more likely to have type 2 diabetes than type 1 diabetes and were more likely than White youth to have type 2 diabetes. Studies^11,18^ in other countries have also found that youths from racially oppressed groups have more youth-onset type 2 diabetes. In addition to being less financially capable, the living environment, educational attainment, and job opportunities for families from minoritized groups are mostly worse than those for White families.^19^ Studies show high rates of adolescent type 2 diabetes in other Indigenous populations.^20^ Poverty is a common phenomenon among families of racially oppressed or Indigenous populations.^11^ In China and India, there was a phase of unfavorable economic conditions characterized by rapid postnatal catch-up growth, resulting in an increase in adolescents with type 2 diabetes.^20^ Some studies^12,21,22^ have confirmed that poverty is associated with the development and complications of type 2 diabetes in adults. Few studies^3,5^ have examined poverty as a risk factor for developing type 2 diabetes in youth. Our study found that low family income was associated with a significantly higher hazard of youth-onset type 2 diabetes compared with high family income. The association seems to have a dose-dependent relationship, implying that the lower the household income, the higher the risk of type 2 diabetes in adolescents. Other studies^23^ have also found a dose-dependent relationship between poverty and health status. In addition, we observed consistent results when we restricted our study participants to children between the ages of 7 and 19 years. Our research also showed a significant association between children and adolescents from lower-income households and a higher risk of hospitalization for diabetes complications.

Possible explanations for the link between poverty and youth-onset type 2 diabetes are that young people from very-low-income families may experience food insecurity. They may buy cheaper, unhealthy, high-calorie foods or sugar-sweetened beverages.^22^ Second, family members of adolescents from economically disadvantaged families may have lower levels of education and unhealthy lifestyles, such as physical inactivity, poor sleep habits, smoking, or binge drinking. Young people exposed to unhealthy lifestyles during critical life course may be more susceptible to developing type 2 diabetes.^22,24^ Third, poverty may increase the risk of type 2 diabetes in adults, and parents with diabetes are more likely to have children with type 2 diabetes.^4,9,21,25^ Fourth, research has shown that psychological or occupational stress of poverty in adults leads to type 2 diabetes. Adolescence is associated with psychological challenges; the stress accompanying poverty may lower resilience in adolescents.^23,26^ The cumulative effect of socioeconomic stressors during the critical period of adolescence may induce proinflammatory responses and physiological changes that lead to type 2 diabetes.^23^

Our study also found that adolescents with older age, female sex, overweight or obesity, dyslipidemia, gout, and psychiatric disorders had a higher hazard of youth-onset type 2 diabetes. Previous studies^3,5^ have shown that age and being female are factors associated with increased risk of developing type 2 diabetes in adolescents. As a result of the change in eating habits, some adolescents consume large quantities of sugar-sweetened beverages and high-calorie foods, leading to increased obesity, which is closely related to youth-onset type 2 diabetes.^5,7,27^ Adolescents with type 2 diabetes have a higher probability of metabolic syndromes, such as dyslipidemia and gout, than those without type 2 diabetes.^3,4,28^ Adolescent rebellion due to academic or family pressure may result in anxiety, depression, and other psychiatric disorders among adolescents with type 2 diabetes.^4,9,26^

Adolescents have a low risk of comorbidities and low mortality rates.^29^ However, our study found that children and adolescents from poorer families had a significantly higher hazard of premature death than those from higher-income families. Previous studies have also found that adolescents from financially disadvantaged families have higher mortality rates.^19,23^ It is possible that these adolescents live in a poor environment, often consume unhealthy foods, use tobacco and alcohol, avoid exercise, and receive relatively little medical care, which leads to a higher risk of mortality.

Our study has several clinical implications. First, children and adolescents from economically deprived families had a higher hazard of youth-onset type 2 diabetes, thus emphasizing the need for family education to promote healthy eating behavior (restricted use of high-calorie foods and sugary beverages), physical activity, and limited tobacco use to reduce the risk of type 2 diabetes. Second, young people from poor families should undergo regular evaluations of blood glucose and body weight. High blood glucose or continuous weight gain needs active intervention to reduce the incidence of youth-onset type 2 diabetes. Third, children and adolescents with type 2 diabetes from economically disadvantaged families should be encouraged toward regular follow-ups, medication adherence, and optimal control of blood glucose to reduce future complications of diabetes.

### Limitations

There were some limitations to this research. First, this national health data set lacked complete information on dietary habits and physical activity, which could affect the results of this study. However, we adjusted for sex, age, comorbidities, and number of outpatient visits per year to minimize confounding. This database does not include family history information, so we are unable to assess the impact of sibling clustering within a family, which could inflate estimates of outcome. Second, overweight, obesity, and severe obesity were defined by *ICD-9* and *ICD-10-CM* diagnosis, which would underestimate the prevalence of these conditions. However, they may have high specificity. Third, biochemical tests for hemoglobin A_1C_, glucose, renal function, and cholesterol levels were unavailable from this data set, which prevented accurate diagnosis of diabetes and assessment of other conditions in patients. However, we used *ICD-9* and *ICD-10-CM* codes to identify type 2 diabetes and comorbidities. The *ICD-9* and *ICD-10-CM* coding algorithm that defined type 2 diabetes was validated with good accuracy. This database lacked information on insulin levels, islet cell antibodies, insulin antibodies, glutamic acid decarboxylase antibodies, and genetic testing. Some young people with type 1 diabetes, latent autoimmune diabetes in adults, and monogenic diabetes could be misdiagnosed with type 2 diabetes in youth. However, we excluded patients taking continuous insulin therapy within 3 months of diabetes diagnosis to exclude possible type 1 diabetes. Fourth, household income was a reliable indicator of socioeconomic status in our database. We did not use education attainment and occupation to assess outcomes because compulsory education in Taiwan is up to high school, and these adolescents received free education up to the high school level. Moreover, most of these adolescents would be in school, not the workforce. Fifth, the results may not apply to other ethnic groups because the study involved a Chinese population. However, our study may provide valuable information on Asian populations. Sixth, observational studies usually have some unknown residual confounding factors; therefore, the results reveal an association between the factors, not a causal relationship. Randomized clinical trials are needed to verify our findings.

## Conclusions

This nationwide, population-based, cohort study found that young people from low-income families had a significantly higher hazard of outpatient and inpatient diagnosed type 2 diabetes in youth and mortality than those from high-income families. Further research to reveal the factors underlying this association may improve the accuracy of identifying individuals at greatest risk for developing type 2 diabetes in youth.

## Notes

Supplement 1. **eAppendix.** Defining Type 2 and Type 1 Diabetes **eTable 1.** Diseases and Associated *ICD-9* and *ICD-10* Codes **eTable 2.** Factors for All-Cause Mortality and Risk of Diabetes Incidence in Children and Adolescents Aged 0 to 19 Years in 2008 in Taiwan **eTable 3.** Risk of Diabetes Incidence and Hospitalization for Diabetes Among Children and Adolescents Aged 7 to 19 Years in 2008 in Taiwan by Family Income Group **eFigure.** Case Selection Flow Chart

Supplement 2.
Data Sharing Statement
