# Supplementary material for: Parental Income Level and Risk of Developing Type 2 Diabetes in Youth
Source: JAMA Netw Open. 2023 Nov 30;6(11):e2345812. doi: 10.1001/jamanetworkopen.2023.45812 (PMC10690454; doi:10.1001/jamanetworkopen.2023.45812)
Supplement: Supplement 1. — eAppendix. Defining Type 2 and Type 1 Diabetes eTable 1. Diseases and Associated ICD-9 and ICD-10 Codes eTable 2. Factors for All-Cause Mortality and Risk of Diabetes Incidence in Children and Adolescents Aged 0 to 19 Years in 2008 in Taiwan eTable 3. Risk of Diabetes Incidence and Hospitalization for Diabetes Among Children and Adolescents Aged 7 to 19 Years in 2008 in Taiwan by Family Income Group eFigure. Case Selection Flow Chart [file jamanetwopen-e2345812-s001.pdf]

Supplemental Online Content

Yen FS, Wei JCC, Liu JS, Hwu CM, Hsu CC. Parental income level and risk of developing type 2 diabetes in youth. *JAMA Netw Open*. 2023;6(11):e2345812. doi:10.1001/jamanetworkopen.2023.45812

eAppendix. Defining Type 2 and Type 1 Diabetes
eTable 1. Diseases and Associated *ICD-9* and *ICD-10* Codes
eTable 2. Factors for All-Cause Mortality and Risk of Diabetes Incidence in Children and Adolescents Aged 0 to 19 Years in 2008 in Taiwan
eTable 3. Risk of Diabetes Incidence and Hospitalization for Diabetes Among Children and Adolescents Aged 7 to 19 Years in 2008 in Taiwan by Family Income Group
eFigure. Case Selection Flow Chart

This supplemental material has been provided by the authors to give readers additional information about their work.

© 2023 Yen FS et al. *JAMA Network Open.*

eAppendix. Defining Type 2 and Type 1 Diabetes

Type 2 diabetes

Definition

ICD-9-CM: 250.xx, except 250.1x; ICD-10-CM: E11, with ≥3 outpatient visits or ≥1 inpatient admission for T2D within 1 year.

Performance measures

Sensitivity: 90.9%

Specificity: 94.9%

Positive predictive value: 92.0%

To increase the accuracy of this algorithm, we excluded children or adolescents on insulin therapy within 3 months of diabetes diagnosis or if they received treatment in an emergency room or hospital for diabetic ketoacidosis.

Source

Hsieh MT, Hsieh CY, Tsai TT, et al. Validation of Stroke Risk Factors in Patients with Acute Ischemic Stroke, Transient Ischemic Attack, or Intracerebral Hemorrhage on Taiwan's National Health Insurance Claims Data. Clin Epidemiol. 2022;14:327-335. doi: 10.2147/CLEP.S353435

Type 1 diabetes

Definition

© 2023 Yen FS et al. *JAMA Network Open.*

ICD-9-CM: 250.1x; ICD-10-CM: E10, with ≥1 inpatient admission, or ≥3 outpatient visits for type 1 diabetes within 1 year, and was registered in the Catastrophic Illness Database.

Performance measures

Positive predictive value: 98.3%

In Taiwan, people with type 1 diabetes are required to compile various documents, including a physician's diagnosis, relevant medical records, test results, fasting or glucagon-stimulated C-peptide levels, anti-glutamic acid decarboxylase (GAD) antibody levels, and any history of diabetic ketoacidosis. These documents must be submitted to the National Health Insurance Administration (NHIA) for evaluation and endorsement for a catastrophic illness card. Possession of this card entitles patients to exemption from co-payments for medical treatment and prescriptions.

Source

Lin WH, Wang MC, Wang WM, et al. Incidence of and mortality from Type I diabetes in Taiwan from 1999 through 2010: a nationwide cohort study. PLoS One. 2014;9(1):e86172. doi: 10.1371/journal.pone.0086172

© 2023 Yen FS et al. *JAMA Network Open.*

eTable 1 — Diseases and Associated *ICD-9* and *ICD-10* Codes

Disease	ICD-9-CM codes	ICD-10-CM codes
Type 2 diabetes	250.xx, except 250.1x	E11
Type 1 diabetes	250.1x	E10
Dialysis	V56.0, V56.8, V45.1, V45.11	Z49.31, Z49.32, Z99.2
Overweight	278.02, 783.1, V85.2	R63.5
Obesity	278.00, 649.1, V77.8, V85.3	E66.09, E66.1, E66.8, E66.9, Z13.89
Severe obesity	278.01, 649.2, V45.86, V85.4	E66.01, E66.2
Smoking status	305.1, 649.0, V15.82	F17.200, F17.201, F17.210, F17.220, F17.221, F17.290, F17.291, Z87.891
Alcohol-related disorders	291, 303, 305.0, 571.0-571.3, V11.3, V79.1	F10, K70.40, K70.41, K70.9
Hypertension	401–405, A26	I10, I11, I12, I13, I15, N26
Dyslipidemia	272	E71.30, E71.31, E71.32, E71.39, E75.21, E75.22, E75.23, E75.24, E75.25, E75.29, E75.3, E75.4, E75.5, E75.6, E77, E78.0, E78.1, E78.2, E78.3, E78.4, E78.5, E78.6,

© 2023 Yen FS et al. *JAMA Network Open.*

		E78.70, E78.71, E78.72, E78.79, E78.8, E78.9
Gout	274, V77.5	M10, M1A
Psychiatric disorders	291–299	F10-F59, F90-F99

© 2023 Yen FS et al. *JAMA Network Open.*

eTable 2 — Factors for All-Cause Mortality and Risk of Diabetes Incidence in Children and Adolescents Aged 0 to 19 Years in 2008 in Taiwan

	**All-cause mortality**		**Diabetes incidence**	
	**Adjusted hazard ratio (95% confidence intervals)**	**P-value**	**Adjusted hazard ratio (95% confidence intervals)**	**P-value**
Family income groups				
Very low	2.18 (1.97-2.41)	<0.001	1.55 (1.41-1.71)	<0.001
Low	1.51 (1.42-1.60)	<0.001	1.34 (1.27-1.41)	<0.001
Middle	1.22 (1.14-1.31)	<0.001	1.27 (1.20-1.34)	<0.001
High	Reference		Reference	
Age, per years	1.08 (1.07-1.08)	<0.001	1.08 (1.08-1.09)	<0.001
Male vs. female	1.79 (1.72-1.85)	<0.001	0.97 (0.94-0.99)	0.045
Overweight	1.69 (0.63-4.56)	0.30	0.58 (0.38-0.87)	0.008
Obesity	1.33 (0.91-1.94)	0.15	5.17 (4.5-5.94)	<0.001
Severe obesity	0.90 (0.37-2.19)	0.82	2.64 (1.87-3.73)	<0.001
Smoking	3.11 (0.78-12.44)	0.11	1.38 (0.19-9.80)	0.74
Alcohol-related disorders	4.25 (1.59-11.36)	0.004	1.40 (0.45-4.36)	0.56
Hypertension	1.62 (1.11-2.36)	0.012	1.17 (0.92-1.49)	0.21

© 2023 Yen FS et al. *JAMA Network Open.*

Dyslipidemia	3.13 (2.35-4.17)	<0.001	3.82 (3.22-4.55)	<0.001
Gout	0.79 (0.56-1.12)	0.19	1.95 (1.61-2.35)	<0.001
Psychiatric disorders	2.78 (2.44-3.17)	<0.001	2.18 (1.92-2.48)	<0.001

Model adjusted age, sex, overweight, obesity, severe obesity, smoking, alcohol, hypertension, dyslipidemia, gout, psychotic disorders, CCI scores, and frequency of outpatient visits per year.

© 2023 Yen FS et al. *JAMA Network Open.*

eTable 3. Risk of Diabetes Incidence and Hospitalization for Diabetes Among Children and Adolescents Aged 7 to 19 Years in 2008 in Taiwan by Family Income Group

	Case	Incidence rate per 1000 person-years	Crude model		Adjusted model	
			Hazard ratio (95% confidence intervals)	p-value	Hazard ratio (95% confidence intervals)	P-value
Diabetes incidence						
Family income groups						
Very low	499	0.55	2.19 (1.97-2.43)	<0.001	2.18 (1.96-2.43)	<0.001
Low	6681	0.37	1.51 (1.42-1.61)	<0.001	1.51 (1.42-1.62)	<0.001
Middle	2278	0.29	1.23 (1.14-1.32)	<0.001	1.25 (1.16-1.34)	<0.001
High	1075	0.24	Reference		Reference	
Hospitalization for diabetes complications						
Very low	91	0.1	2.40 (1.86-3.09)	<0.001	2.33 (1.81-3.01)	<0.001
Low	1304	0.07	1.75 (1.50-2.05)	<0.001	1.81 (1.54-2.11)	<0.001
Middle	428	0.06	1.39 (1.16-1.65)	<0.001	1.40 (1.17-1.67)	<0.001

© 2023 Yen FS et al. *JAMA Network Open.*

High	177	0.04	Reference	Reference

Model adjusted age, sex, overweight, obesity, severe obesity, smoking, alcohol, hypertension, dyslipidemia, gout, psychiatric disorders, CCI scores, and frequency of outpatient visits per year.

© 2023 Yen FS et al. *JAMA Network Open.*

eFigure — Case Selection Flow Chart

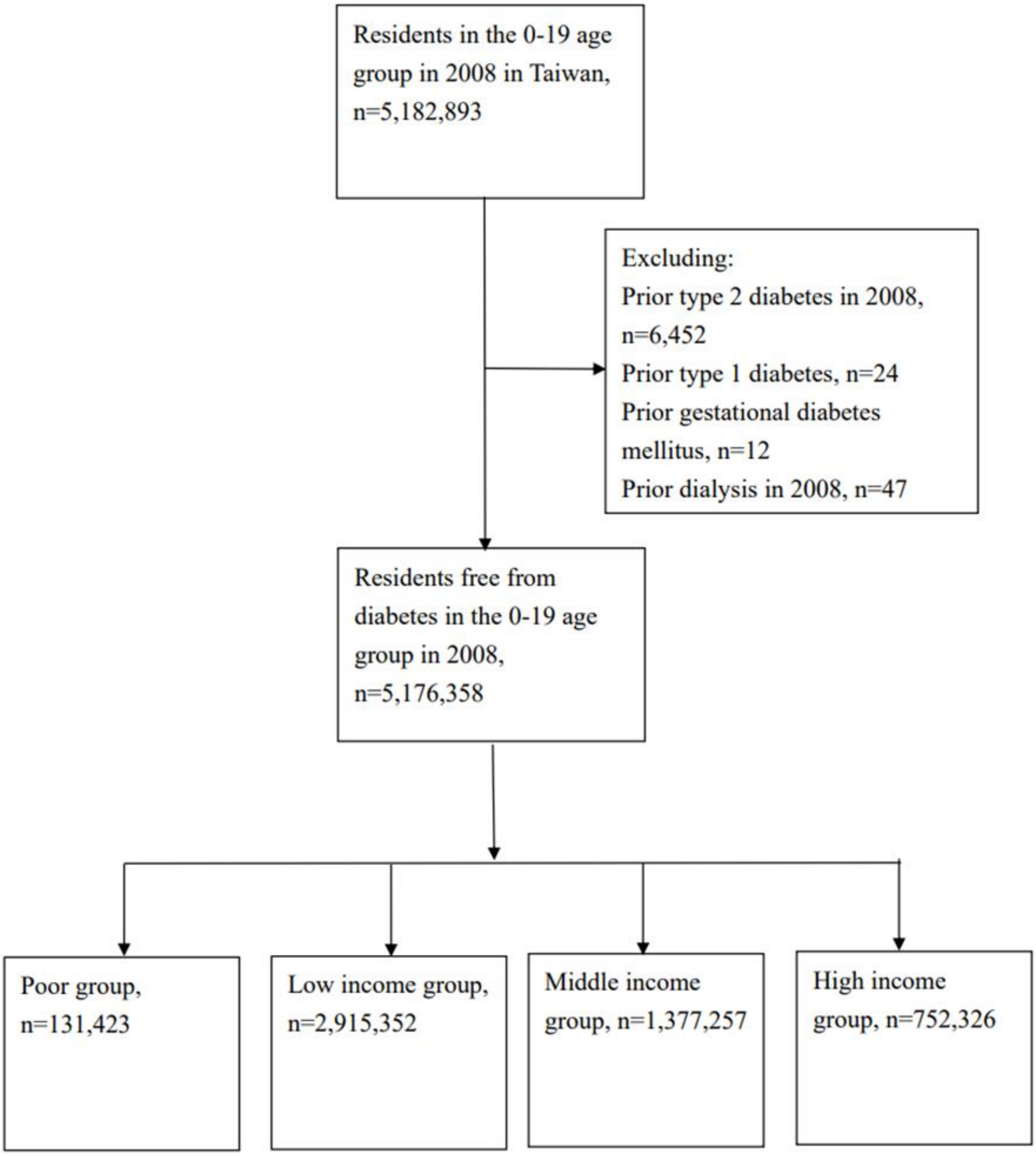

© 2023 Yen FS et al. *JAMA Network Open.*
